# Endemic Tularemia, Sweden, 2003

**DOI:** 10.3201/eid1109.041189

**Published:** 2005-09

**Authors:** Lara Payne, Malin Arneborn, Anders Tegnell, Johan Giesecke

**Affiliations:** * European Programme for Intervention Epidemiology Training, Solna, Sweden;; † Swedish Institute for Infectious Disease Control, Solna, Sweden;; ‡ National Board of Health and Welfare, Stockholm, Sweden

**Keywords:** Tularemia, endemic infection, Sweden, outbreak, dispatch

## Abstract

Tularemia cases have been reported in Sweden since 1931, but no cyclical patterns can be identified. In 2003, the largest outbreak of tularemia since 1967 occurred, involving 698 cases. Increased reports were received from tularemia-nonendemic areas. Causal factors for an outbreak year and associated geographic distribution are not yet understood.

The ability of *Francisella tularensis*, the bacterial pathogen of tularemia, to infect at low levels and cause a high prevalence of illness and death in humans (*1*) has led to its inclusion in the ranks of potential bioterrorism agents (*2*). However, endemic forms of a less virulent subspecies of the agent also exist in parts of the Northern Hemisphere. Bioterrorism concerns further the need for surveillance of tularemia-endemic areas of the world, both to learn more about the disease and to differentiate natural from deliberate outbreaks (*3*). Sweden has had reported cases of tularemia since 1931, with outbreaks of variable magnitude, but with no cyclical patterns or trends (*4*). In 2003, 698 cases of tularemia were reported, the highest number since 1967 (Figure 1); this outbreak was larger than those usually observed (100–500 cases). Increased numbers of cases were also reported as acquired outside the identified tularemia-endemic region, similar to the situation in the 2000 outbreak (*5*). This article describes the epidemiology of cases in 2003.

**Figure 1:**
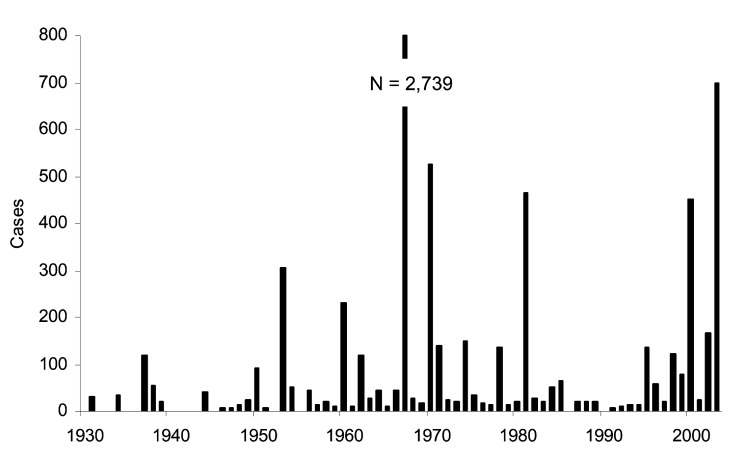
Number of tularemia cases reported in Sweden by year (1930–2003).

## The Study

Tularemia has been a notifiable disease in Sweden since 1968. Clinicians report diagnoses of tularemia, with or without laboratory evidence, to the county medical officer (CMO) and the Department of Epidemiology, Swedish Institute for Infectious Disease Control (EPI/SMI). Reports from regional hospital microbiology laboratories of positive cases of tularemia are also sent to SMI and CMOs and are matched to clinical reports, on the basis of a unique national personal identifying number. For this analysis, we included all cases reported to SMI and registered in the database between January 1 and December 31, 2003.

Of the 698 cases reported in 2003, 591 were diagnosed in 2003, of which 567 were reported to have been acquired in Sweden (8 cases in Finland, 1 in Turkey, 15 not known). A sharp peak of cases was observed in August, with cases tapering off until December (Table 1). More male patients (322, 57%) were reported than female patients, and the 45- to 64-year age group was the most affected in both sexes (Table 2).

**Table 1:** Tularemia cases acquired and reported in Sweden in 2003 and in 2000, by month of diagnosis

Month	No. 2003 cases (N = 567)	No. 2000 cases (N = 384)
January	0	0
February	1	0
March	1	1
April	2	0
May	0	0
June	1	0
July	51	1
August	300	53
September	164	210
October	39	98
November	7	20
December	1	1

**Table 2:** Tularemia cases by age group and sex, Sweden, 2003

Age group (y)	No. females (n = 245)	No. males (n = 322)	Total (%) (n = 567)
<6	8	11	19 (3)
7–17	23	27	50 (9)
18–24	4	10	14 (2)
25–44	58	81	139 (25)
45–64	117	151	268 (47)
65–79	31	36	67 (12)
>80	4	6	10 (2)

Of 522 cases that had a known route of infection, animal contact or insect bite was most common (n = 475, 91.0%), and 23 cases (4.4%) were reported to have been acquired by inhaling contaminated dust or other material. The remaining reports were of infection acquired by another route (n = 22, 4.2%) or associated with work (n = 2, 0.4%). Possible exposure risks of farm or outdoor work were mentioned in an additional 25 cases, berry or mushroom picking in 5 cases, and outdoor pursuits, such as golf or fishing, in 6 cases. Clinical information about a tularemia case is not systematically collected at the national level, but the oropharyngeal form of tularemia was mentioned in 1 case. This patient had acquired infection in the northern coastal area of Sweden.

**Figure 2:**
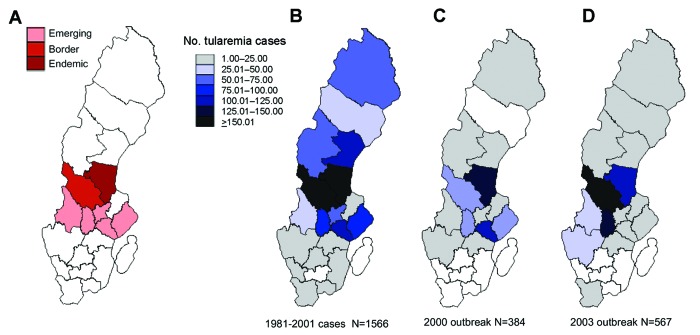
Tularemia cases by county of probable infection, Sweden. A) Areas in analysis by Eliasson et al. B) 1981–2001 cases (N = 1,566). C) 2000 outbreak (N = 384). D) 2003 outbreak (N = 567). White areas indicate no reports.

Overall, reports were received concerning infections acquired in 15 of the 21 counties in Sweden (Figure 2). Only 22% of patients in 2003 seemed to have been infected in what is considered the disease-endemic area of central Sweden, and as many as 67% were infected in border or disease-emerging areas (*5*). The main observations from the largest outbreak of tularemia in Sweden since 1967 were that cases occurred earlier in the year, in greater numbers outside the disease-endemic area in Sweden, affected those 45–64 years of age most frequently, and were mainly acquired through animal contact or insect bites.

We expected that the reporting of tularemia cases for surveillance would be fairly complete because tularemia is a notifiable disease in Sweden, and the public is interested in the infection. An assessment of reporting completeness to SMI identified 98.5% completeness (range 84.6%–99.6%) for tularemia reports (1998–2002) (*6*). However, mild or even undiagnosed cases are likely to be missed by the reporting system.

The appearance of cases in midsummer is earlier than in the 2000 outbreak (Table 1). Previous outbreaks have usually began in late summer or early autumn (*4*). Since >90% of cases reported infection through insect bites or animal contact, this seasonal shift may be linked to climatic or ecologic factors in a particular year. Cases are registered in the SMI database by date of reporting and not date of diagnosis. However, median delay between date of sample collection and date of reporting for the period 1998 to 2002 was 11 days (interquartile range 8–19 days) (*7*). Therefore, the seasonality observed in the epidemic curve of 2003 cases was likely not greatly affected by possible reporting delay.

## Conclusions

Responses regarding the route of infection may be biased because many persons in Sweden associate tularemia with mosquito bites. Nonetheless, the seasonal distribution of cases observed would support this as a route of infection. The age and sex distribution did not differ from those of the last large outbreak in 2000 and likely reflect the age and sex distribution of persons working outdoors in farms or gardens in rural areas.

Sporadic tularemia cases outside of the disease-endemic north-central region and northern coastal region have been recorded since 1931 in Sweden. As in the 2000 outbreak, cases were reported from counties that previously had very few reports (*4*). However, even more reports were received in 2003 than in 2000 of infections acquired outside the tularemia-endemic area (427 [78%] of 544 cases in 2003 vs. 227 [61%] of 370 in 2000, chi-square test = 31.79, p<0.001; Figure 2). The geographic distribution of tularemia seems to be changing. Public awareness and changes in behavior for seeking medical care could contribute to such a change, but no evidence suggests that these factors differ from those in previous years.

The factors affecting the likelihood of an outbreak year remain unknown, principally because the natural reservoir of infection has yet to be identified (*8*). After the outbreak in Sweden in 2000, a case-control study found significant associations between acquiring tularemia and being bitten by a mosquito, doing farm work, and owning a cat (*5*). The role of ticks (*9*) and mosquitoes as potential vectors for tularemia needs further investigation. Despite the interest and awareness of tularemia in Sweden, much remains to be understood about the dynamics of this infection among reservoir, vector, and human populations.
